# Biliary sludge and recurrent ketoacidosis: a case report

**DOI:** 10.1186/1758-5996-1-28

**Published:** 2009-12-22

**Authors:** Sanjay Kalra, Bharti Kalra, Anuj Thakur, Amit Sharma

**Affiliations:** 1 Department of Endocrinology, Bharti Hospital, Karnal, India; 2 Department of Diabetology, Bharti Hospital, Karnal, India; 3 Department of Neonatology, Thakur Nursing Home, Karnal, India; 4 Department of Clinical Research, Bharti Hospital, Karnal, India

## Abstract

A five year old boy, weighing 14 kg with no family history of diabetes, presented in frank diabetic ketoacidosis. He recovered, but continued to have episodes of ketoacidosis. He was diagnosed to have biliary sludge, which recovered with insulin treatment.

## Case Report

A five year old boy, 104 cm tall, weighing 14 kg with no family history of diabetes, presented in frank diabetic ketoacidosis. The parents reported history of weight loss, polydipsia, polyuria and polyphagia for two weeks. On persistent questioning, they admitted history of low grade fever and episodic mild abdominal pain for the past one month.

On examination, he had rachitic features, and abdomen was protuberant. No evidence of pallor, fever, icterus, lymphadenopathy, Bitot's spots was noted. Dental age was 5 years. There was mild epigastric tenderness, with no radiation to back, or change on posture. Liver span was 8 cm, and spleen was not palpable.

Ketosis was corrected within 24 hours with 18 units regular aspart insulin, and the patient discharged after 72 hours on a 3-dose regime (regular aspart - regular aspart - premixed aspart) totaling 19 units/day.

He returned after 3 days with moderate ketonuria, but no evidence of acidosis. The parents were adamant that he had followed the prescribed diet pattern and not missed any insulin dose.

Two such episodes recurred within two weeks, each accompanied by severe upper abdominal pain, and treated successfully by IV insulin infusion over 12-24 hours.

On detailed investigation, ultrasonography revealed multiple gall stones. The liver echotexture and size was normal. This finding was confirmed a week later. A stool examination was negative for parasitic ova/cysts. A complete hemogram showed no evidence of hemolysis. Fasting lipid profile, serum amylase and hepatic function tests were normal, except for a raised serum alkaline phosphatase (347 IU/ml [normal range 150-250 IU/ml]). A Widal test was non-reactive, and blood culture was not done.

After initial difficulties for the first 2 weeks, the patient stabilized on 15 units/day of aspart insulin, in 3 divided doses. Frequent abdominal pain prompted a surgical referral for cholecystectomy, with the diagnosis of cholelithiasis, but a pre operative scan (done two weeks after the first scan,) revealed no abnormality. The earlier finding was retrospectively thought to be biliary sludge, as cholelithiasis would not have been self-limiting.

## Discussion

Adult patients of diabetes frequently get decompensated because of cholecystitis associated with cholelithiasis. However, biliary sludge is not infrequent finding on ultrasonography in patients with chronic infection such as typhoid, and disappears in a few weeks. Biliary sludge has also been reported with intensive insulin therapy [1,2].

Biliary sludge or acute acalculous cholecystitis has been reported in pediatric age group by other authors [3,4], but none has highlighted the association of this clinical entity with type 1 diabetes mellitus, diabetic ketoacidosis or recurrent ketonuria.

This case reports highlights the occurrence of biliary sludge in paediatric patients of diabetes. Biliary sludge should be ruled out in any type 1 diabetic patient presenting with recurrent ketosis. Biliary sludge should be considered in any patient with vague abdominal symptoms, and different findings on ultrasound done at different points of time.

Biliary sludge may be managed non-surgically, as in this case, by optimal management of diabetes. This case also highlights the need to continue conservative management, and not rush for surgery in patients with suspected cholelithiasis.

## Consent

Written informed consent was obtained from the patient for publication of this case report and accompanying images. A copy of the written consent is available for review by the Editor-in-Chief of this journal.

## Competing interests

The authors declare that they have no competing interests.

## Authors' contributions

SK, BK and AT managed the patient, with SK providing endocrine care, and AT providing pediatric care. All authors contributed to paper writing and read the final manuscript.
